# Antigenic cartography of SARS-CoV-2 reveals that Omicron BA.1 and BA.2 are antigenically distinct

**DOI:** 10.1126/sciimmunol.abq4450

**Published:** 2022-06-23

**Authors:** Anna Z. Mykytyn, Melanie Rissmann, Adinda Kok, Miruna E. Rosu, Debby Schipper, Tim I. Breugem, Petra B. van den Doel, Felicity Chandler, Theo Bestebroer, Maurice de Wit, Martin E. van Royen, Richard Molenkamp, Bas B. Oude Munnink, Rory D. de Vries, Corine GeurtsvanKessel, Derek J. Smith, Marion P. G. Koopmans, Barry Rockx, Mart M. Lamers, Ron Fouchier, Bart L. Haagmans

**Affiliations:** ^1^ Viroscience Department, Erasmus Medical Center, Rotterdam, Netherlands.; ^2^ Department of Neurology, Erasmus University Medical Center, Rotterdam, Netherlands; ^3^ Department of Pathology, Erasmus University Medical Center, Rotterdam, Netherlands.; ^4^ Center for Pathogen Evolution, Department of Zoology, University of Cambridge, Cambridge, CB2 3EJ, UK

## Abstract

The emergence and rapid spread of SARS-CoV-2 variants may impact vaccine efficacy significantly. The Omicron variant termed BA.2, which differs substantially from BA.1 based on genetic sequence, is currently replacing BA.1 in several countries, but its antigenic characteristics have not yet been assessed. Here, we used antigenic cartography to quantify and visualize antigenic differences between early SARS-CoV-2 variants (614G, Alpha, Beta, Gamma, Zeta, Delta and Mu) using hamster antisera obtained after primary infection. We first verified that the choice of the cell line for the neutralization assay did not affect the topology of the map substantially. Antigenic maps generated using pseudotyped SARS-CoV-2 on the widely used VeroE6 cell line and the human airway cell line Calu-3 generated similar maps. Maps made using authentic SARS-CoV-2 on Calu-3 cells also closely resembled those generated with pseudotyped viruses. The antigenic maps revealed a central cluster of SARS-CoV-2 variants, which grouped based on mutual spike mutations. Whereas these early variants are antigenically similar, clustering relatively close to each other in antigenic space, Omicron BA.1 and BA.2 have evolved as two distinct antigenic outliers. Our data show that BA.1 and BA.2 both escape vaccine-induced antibody responses as a result of different antigenic characteristics. Thus, antigenic cartography could be used to assess antigenic properties of future SARS-CoV-2 variants of concern that emerge and to decide on the composition of novel spike-based (booster) vaccines.

## Teaser

Antigenic evolution by SARS-CoV-2 can be monitored by antigenic cartography, informing future coronavirus vaccine strain selections.

## INTRODUCTION

Since its emergence in Wuhan, China, in 2019, severe acute respiratory syndrome coronavirus 2 (SARS-CoV-2) has caused over 500 million cases and 6.3 million deaths (as of June 2022) (*1*). The initial virus that spread globally was characterized by a D614G change in the spike (S) protein (*2*). Approximately one year into the pandemic other variants with 6-12 mutations in the S protein started to become dominant in various countries (*3*). These variants included the Alpha variant in the United Kingdom, the Beta variant in South Africa, and the Gamma variant in Brazil, of which Alpha became the dominant variant globally by early 2021. In the summer of 2021, the Delta variant emerged first in India, and replaced Alpha globally within several months (*4*–*7*). Emerging variants are termed Variants of Concern (VOC) by the World Health Organization (WHO) if they are associated with a major change in epidemiology and/or clinical presentation, increased virulence, increased transmissibility, and/or decreased effectiveness of public health and social measures or available diagnostics, vaccines or therapeutics (*8*). In addition, the WHO has designated other variants as Variants of Interest (VOI), which possess mutations predicted or known to affect antibody escape, virulence, or transmission.

At the end of 2021, the VOC Omicron (sublineage BA.1) emerged in Botswana and South Africa, carrying 30 mutations in S, raising concerns for extensive immune evasion (*9*). Whereas several earlier VOCs and VOIs exhibit some levels of antibody escape (e.g., Beta, Gamma, Delta, and Mu), they were still neutralized well by convalescent and post-vaccination sera (*10*–*14*). In contrast, Omicron BA.1 almost completely escapes neutralizing antibodies, leading to low levels of remaining protective antibodies in most previously infected or vaccinated individuals, and a high frequency of breakthrough infections. This antibody escape at least partly explains why this variant has become the dominant variant globally over the span of a few weeks (*15*–*18*). Fortunately, BA.1 appears to be less virulent compared with earlier variants (*19*, *20*). A second Omicron variant (sublineage BA.2), emerged in South Africa around the same time as BA.1 and differs from BA.1 by 40 mutations (including 18 substitutions and 5 indels in S) (*21*). Whereas BA.2 initially was only sporadically detected, it is currently replacing BA.1 in several countries (*22*). Several reports have indicated that BA.2 escapes neutralization by post-vaccination sera and monoclonal antibodies, which may contribute to its rapid spread (*23*–*25*). In addition, BA.2 may be inherently more transmissible or have considerable antigenic differences from BA.1 and earlier variants.

As SARS-CoV-2 continues to evolve and escape neutralizing antibody responses, it is becoming increasingly important to understand the antigenic relationships among variants and the substitutions that underlie antigenic changes. Antigenic cartography is a tool to quantitatively analyze antigenic drift and visualize the emergence of new antigenic clusters, which is why it is used to semiannually inform influenza virus vaccine strain selection (*26*, *27*). Here, we investigated the neutralizing activity of human post-vaccination sera to both Omicron subvariants and applied antigenic cartography to 11 SARS-CoV-2 variants and hamster antisera elicited against eight SARS-CoV-2 variants by primary infection.

## RESULTS

### Neutralizing activity of human sera against Omicron BA.1 and BA.2

**Fig. 1.**
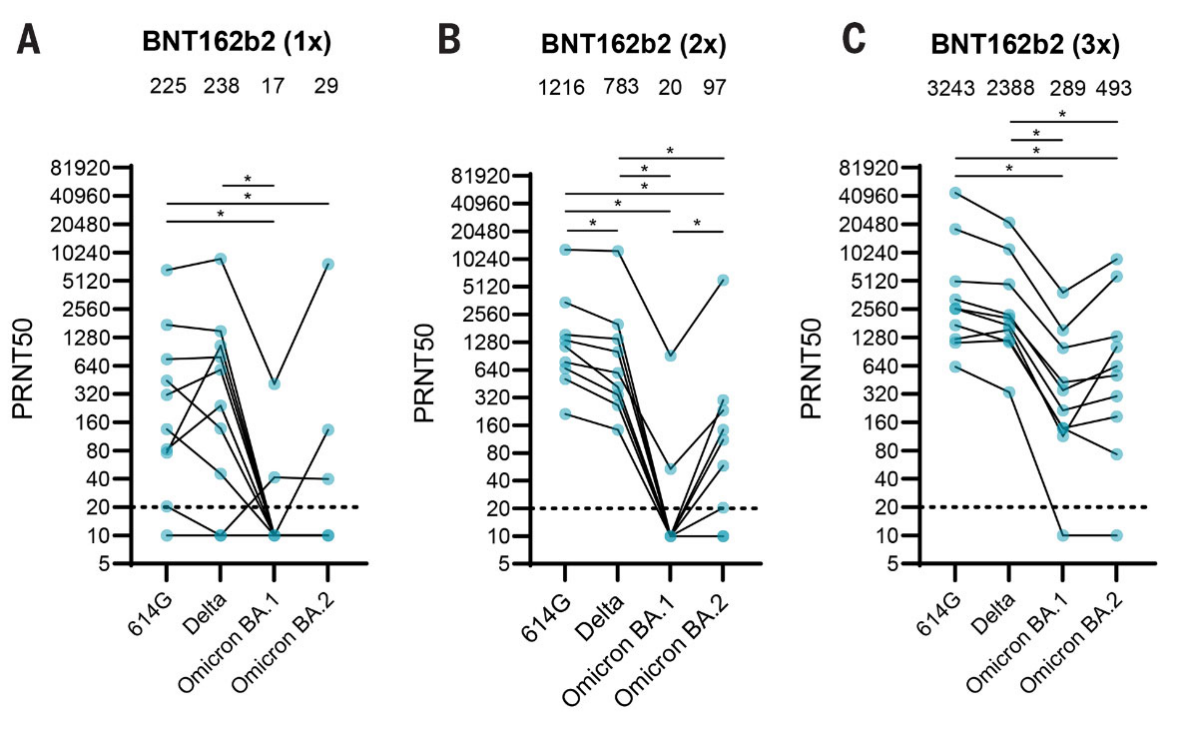
Neutralizing activity of human post-vaccination sera against Omicron BA.1 and BA.2. (**A-C**) Neutralization titers against 614G, Delta, Omicron BA.1 and Omicron BA.2 of vaccinated individuals after vaccination with one (**A**), two (**B**) or three (**C**) doses of BNT162b2. Geometric mean is displayed above each graph. PRNT50: plaque reduction neutralization titers resulting in 50% plaque reduction. Dotted lines indicate limit of detection. One way ANOVA was performed for statistical analysis. *p<0.05. N=10.

Multiple studies have shown that Omicron BA.1 efficiently evades antibody responses post-infection and post-vaccination (*10*–*14*). However, few studies have analyzed antibody responses to Omicron BA.2. Therefore, we investigated to what extent human post-BNT162b2 vaccination sera obtained from ten individuals after one, two or three vaccine doses neutralized Omicron BA.2. Demographic information about these individuals is found in Table S1. After a single BNT162b2 vaccination, on average low neutralizing titers were detected against all variants with a 13- and 8-fold drop in neutralizing titers against Omicron BA.1 and BA.2, respectively, compared with 614G. In line with previous findings, Omicron BA.1 was 61-fold less efficiently neutralized after two BNT162b2 vaccinations (*16*–*18*) (Fig. 1, A and B). In comparison, Omicron BA.2 was neutralized somewhat more efficiently with a 13-fold drop in neutralizing titers compared with 614G. A third vaccination with BNT162b2 reduced the fold change to 614G to 11- and 7-fold for BA.1 and BA.2, respectively. Titers against all variants increased with each dose (Fig. 1, A to C). Combined, these data show that Omicron BA.1 and BA.2 escape antibody responses, and suggest that the height and breadth of the antibody response against SARS-CoV-2 can be increased by repeated exposure to the original antigen. The differential effect of booster vaccination on BA.1 and BA.2 suggested that both variants are antigenically distinct, warranting further analysis, in particular when selecting future vaccine strain candidates. Sera from primary infections with SARS-CoV-2 variants can be used to assess the antigenicity of different variants, however human sera from primary variant infections are increasingly difficult to obtain. We did attempt to obtain human sera post-primary Omicron infection and included ten individuals who had not been vaccinated, two of whom were sampled at two different time points. Four reported a previous SARS-CoV-2 infection. The details of these individuals are found in Table S2. Five individuals had high neutralizing titers against 614G and Delta, suggesting that an additional individual had been infected with another variant prior to their BA.1 infection (Fig. S1A). These sera neutralized both Omicron variants, although to a lesser extent than 614G and Delta (4.3- and 2.2-fold drop in geometric mean titer against BA.1 and BA.2, respectively, when compared to 614G). Of the remaining six sera, four neutralized both BA.1 and BA.2, one individual only neutralized BA.1 and one individual only neutralized 614G. However, all six sera had low neutralizing activity against all variants, including Omicron (geometric mean titers of 14, 32, 63 and 41 against 614D, Delta, Omicron BA.1 and BA.2, respectively) (Fig. S1B). As primary antisera against future variants will become even more difficult to obtain, we also determined the antigenicity of SARS-CoV-2 variants using the Syrian golden hamster model. Hamsters were used as they are highly susceptible to SARS-CoV-2 and are therefore ideal for controlled infections and obtaining well-defined antisera (*28*–*30*).

### Antigenic cartography of SARS-CoV-2

**Fig. 2.**
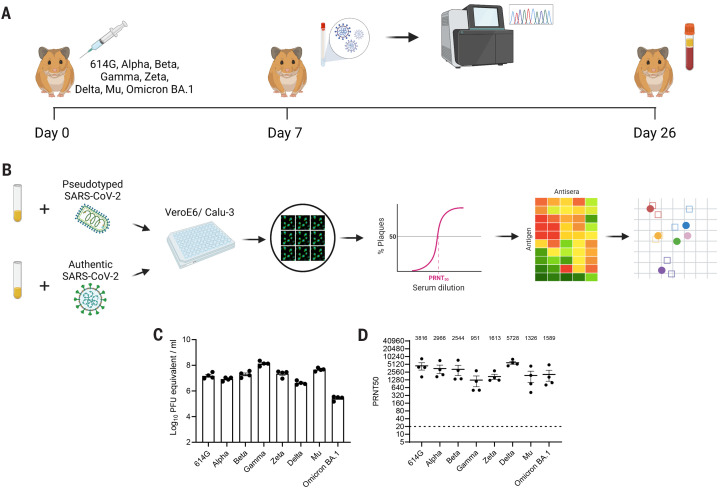
SARS-CoV-2 variants efficiently infect hamsters, inducing high neutralizing antibody titers. (**A**) Hamsters were inoculated with the indicated SARS-CoV-2 variants. Nasal washes were collected 7 days post-infection and sequenced. At 26 days post-infection blood was collected for serological analysis. **(B)** Hamster antisera were assessed for neutralizing antibodies against pseudotyped and authentic SARS-CoV-2. PRNT50 values were used to generate antigenic maps using a multidimensional scaling algorithm. **(C)** RNA titers of nasal washes collected one day post-infection. (**D**) Homologous PRNT50 titers were determined using authentic SARS-CoV-2. Geometric mean is displayed above graph. PRNT50: plaque reduction neutralization titers resulting in 50% plaque reduction. Dotted line indicates limit of detection. Error bars indicate SEM. Panels **A** and **B** were created with BioRender.com.

To investigate the antigenic relationships between BA.1, BA.2 and other SARS-CoV-2 variants, we used antigenic cartography (*31*). We used the Syrian golden hamster model to generate antisera by inoculating hamsters with SARS-CoV-2 variants (614G, Alpha, Beta, Gamma, Zeta, Delta, Mu and Omicron (lineage BA.1)) (Fig. 2A, Fig. S2). Virus stocks and the original respiratory specimens were sequenced to confirm the absence of culture-acquired mutations that may affect antigenic properties. In addition, at seven days post-infection (dpi) swabs were collected and sequenced to confirm that the virus did not change during the experiment. Apart from Delta and Omicron BA.1-infected animals, whose swabs yielded too little virus to sequence, no mutations in spike were detected in swab sequences. Plaque reduction neutralization titers resulting in a 50% reduction in infected cells (PRNT50) obtained with both authentic SARS-CoV-2 and pseudotyped viruses were used to generate antigenic maps (Fig. 2B). All animals were successfully infected, as shown by high viral RNA titers at one dpi in nasal washes and high homologous antibody titers at 26 dpi (Fig. 2, C and D).

Pseudovirus neutralization assays are a safe and widely used tool to assess antibody neutralization. We performed initial neutralization experiments on VeroE6 cells, which are the most commonly used cell line for neutralization assays. We used the spike variants 614D, 614G, Alpha, Beta, Delta, Kappa and Omicron BA.1. All homologous sera neutralized to high titers (Fig. S3, A to H). The lowest cross-neutralizing titers were obtained against Omicron for all sera with 9- to 43-fold reduction compared to homologous neutralization. Sera from Omicron BA.1-infected hamsters poorly neutralized all other variants (2- to 81-fold reduction compared to homologous neutralization). These data show that Omicron BA.1 induces different antibody responses compared with all other variants. Next, we performed pseudovirus neutralization assays on the human airway Calu-3 cell line. In Calu-3 cells SARS-CoV-2 enters using the serine protease-mediated entry pathway that is also used in primary cells, whereas in VeroE6 cells SARS-CoV-2 enters using the cathepsin-mediated endocytic entry pathway (*32*). In addition, the variability in infectivity between variants is lower for Calu-3 cells compared with VeroE6, suggesting that Calu-3 cells may allow for more equal comparisons (*33*). Neutralization titers on Calu-3 cells were similar to VeroE6 and in general correlated well (Fig. S3, I to P, Fig. S4, A to G).

**Fig. 3.**
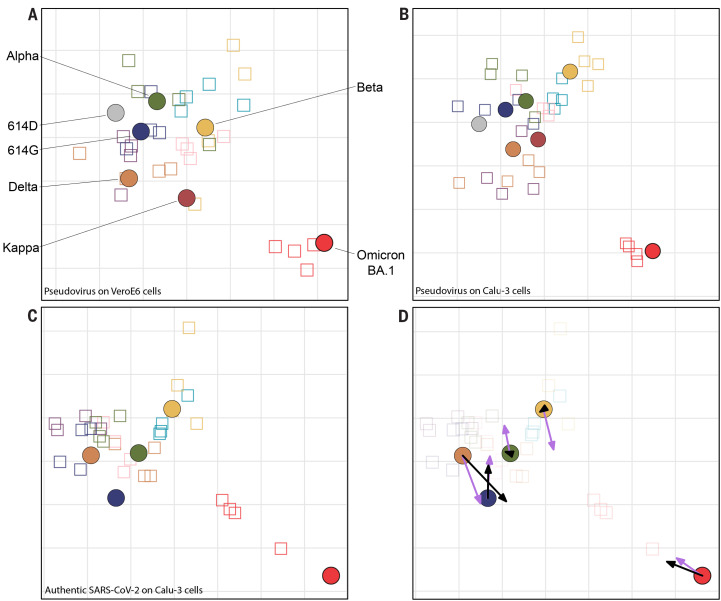
Antigenic maps comparing neutralizations with SARS-CoV-2 pseudoviruses and authentic SARS-CoV-2. (**A-B**) MDS was used to create an antigenic map from the PRNT50 titers generated against 614D, 614G, Alpha, Beta, Delta, Kappa and Omicron pseudoviruses on either VeroE6 (**A**) or Calu-3 (**B**) cells. **(C)** MDS was used to create an antigenic map from the PRNT50 titers generated against 614G, Alpha, Beta, Delta and Omicron authentic SARS-CoV-2. **(D)** Re-display of antigenic map in **C** with lilac arrows indicating antigen positions in map **A** and black arrows indicating antigen positions in map **B**. Viruses are shown as colored circles and antisera as squares with the same outline color as the matching viruses. Viruses and antisera are positioned in the map so that the distances between them are inversely related to the antibody titers, with minimized error. The grid in the background scales to a 2-fold dilution of antisera in the titrations. MDS: multidimensional scaling. PRNT50: plaque reduction neutralization titers resulting in 50% plaque reduction.

Next, we constructed antigenic maps from the neutralization data using a multidimensional scaling (MDS) algorithm described previously (*31*) (Fig. 3, A and B). These were verified to ensure fitted distances correlated well with actual neutralizing titers and to confirm that the data was well represented in two dimensions (Fig. S5, A to D). We found that the map constructed for Calu-3 cells was very similar to the VeroE6 map, since the same antigens plotted within one 2-fold dilution from each other in the two maps (Fig. S6). Therefore, the choice of the cell line for the neutralization assay did not substantially affect the topology of the map. In order to assess whether the map generated with pseudovirus neutralization data would accurately represent authentic SARS-CoV-2, we generated a map with the same antisera as in Fig. 3A and Fig. 3B and 614G, Alpha, Beta, Delta and Omicron viruses on Calu-3 cells. (Fig. 3C). We confirmed that there was a good correlation between the raw neutralizing titers of the 5 variants on Calu-3 and VeroE6 cells (Fig. S7, Fig S8). The map generated with authentic SARS-CoV-2 closely resembled the maps generated with pseudovirus, with the positions of the antigens differing by no more than one 2-fold dilution between maps (Fig. 3D). Antigenically, we found that all variants aside from Omicron BA.1 grouped closely together. Omicron BA.1 formed a distinct antigenic outlier in the map, 10- to 38-fold dilutions away from the nearest virus (Fig. 3, A to C).

Next, we extended the authentic virus dataset to contain a larger set of variants: 614G, Alpha, Beta, Gamma, Zeta, Delta, Delta AY.4.2, Lambda, Mu, Omicron BA.1 and Omicron BA.2.

**Fig. 4.**
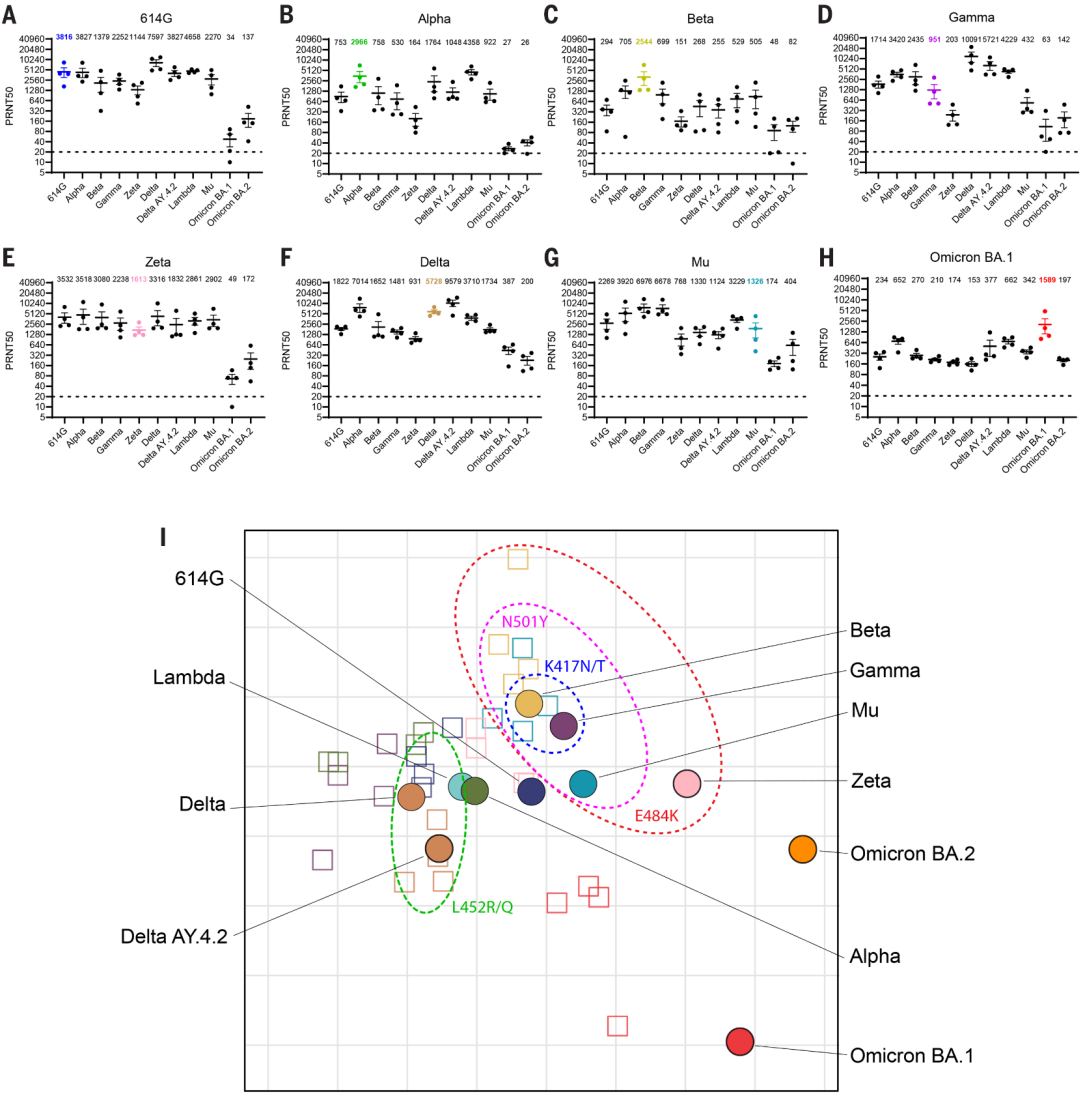
Antigenic cartography using authentic SARS-CoV-2. (**A-H**) Neutralizing titers of hamsters infected with either (**A**) 614G, (**B**) Alpha, (**C**) Beta, (**D**) Gamma, (**E)** Zeta, (**F**) Delta, (**G**) Mu or (**H**) Omicron BA.1 viruses. (**I**) Multidimensional scaling was used to create an antigenic map utilizing PRNT50 titers generated from authentic SARS-CoV-2 on Calu-3 cells. See legend to Fig. 3 for details. Subdivided by dotted ellipses are variants possessing overlapping substitutions as indicated. Geometric mean is displayed above each graph. PRNT50: plaque reduction neutralization titers resulting in 50% plaque reduction. Dotted lines indicate limits of detection. Error bars indicate SEM.

As for pseudotyped virus, homologous sera neutralized to a high titer across all variants (Fig. 4, A to H, homologous titers per panel are a re-display from Fig. 2D). Similar to pseudovirus data, we observed a reduction in neutralization titers of Omicron BA.1 sera against all other variants (2.4- to 9-fold compared to homologous neutralization) and poor neutralization of Omicron BA.1 by all non-homologous sera (8- to 112-fold reduction). In addition, Omicron BA.2 was also poorly neutralized by all sera (7- to 114-fold reduction), including Omicron BA.1 (8-fold reduction). Although Omicron BA.1 and Omicron BA.2 possess many overlapping mutations in S, the differences between the variants were sufficient to prevent efficient cross-neutralization. In agreement, our study shows that antibodies elicited against the original SARS-CoV-2 cluster do not neutralize Omicron BA.1 well, and vice versa.

The antigenic map generated using the extended neutralization dataset shows that, similarly to the maps in Fig. 3, all variants except the Omicron BA.1 and BA.2 group together in one antigenic cluster (Fig. 4I). In line with the limited cross-neutralization of Omicron BA.2 with BA.1 sera, both variants were positioned distantly from each other in the map, with BA.2 somewhat closer to the main cluster than BA.1, indicating that Omicron BA.1 induced qualitatively different antibody responses and BA.1 and BA.2 are antigenically distinct SARS-CoV-2 variants. The antisera corresponding to each virus grouped in the same region of the map, indicating efficient homologous neutralization. As expected, based on Fig. 3, 614G and Alpha are in the center of the cluster. Within this cluster, viruses grouped together based on specific substitutions. Viruses containing E484K (Beta, Gamma, Zeta and Mu) grouped to the top-right of the ancestral 614G virus, whereas viruses containing substitutions L452R/Q grouped to the left of 614G. The Beta and Gamma variants, which in addition to E484K both contain K417N/T also cluster together. The same clustering based on E484K and L452R/Q was observed in the pseudovirus maps.

Next, the antigenic map in Fig. 4I was verified by ensuring fitted distances correlated well with actual neutralizing titers (Fig. S9A). The data was represented well in two dimensions, as increasing to three dimensions did not reduce the error when comparing titers predicted from the map and actual neutralization titers (Fig. S9B). In addition, the relatively small size of the colored regions indicates that the antigens and antisera are well coordinated and positioned confidently in the map (Fig. S9C).

## DISCUSSION

The emergence and rapid spread of the heavily mutated Omicron BA.1 and BA.2 variants suggests that population immunity is exerting strong selective pressure on SARS-CoV-2, favoring the emergence of new antigenic variants. As the number of SARS-CoV-2 variants increases it will become increasingly important to visualize and understand the antigenic relationships between variants. Here, we used antigenic cartography to quantify and visualize SARS-CoV-2 antigenic evolution and demonstrate that Omicron BA.1 and BA.2 have evolved as two antigenically distinct variants, separate from an ancestral cluster with all earlier SARS-CoV-2 variants.

The evolutionary history of SARS-CoV-2 in humans is relatively short compared with viruses that have circulated in humans for decades, such as influenza viruses (*34*). Before the emergence of Omicron, most SARS-CoV-2 variants contained only few substitutions in the S protein and were still recognized by convalescent and post-vaccination sera. These variants all grouped into the same antigenic cluster in our antigenic maps, but within that cluster a grouping could be observed based on S substitutions at position 417, 484 and 452, in line with previous data on human sera (*35*). In their study, Wilks and colleagues used human sera to generate an antigenic map (*35*). Both the maps from our study and the map by Wilks and colleagues show the same clustering of variants, indicating that hamsters and humans generate similar antibody responses. However, differences were observed as well. In the map by Wilks and colleagues, there was approximately a ~2-fold larger distance between 614G and variants Beta, Gamma and Mu, compared to our map containing all authentic viruses. This may be caused by specific antigenic relationships between the large set of variants, as including only 5 variants increased the distance from 614G to Beta by ~2-fold (Fig. 3C). In addition, these differences may be caused by the lower titers observed in naturally-infected humans compared with experimentally-infected hamsters. Low titers against immunizing viruses may drop off more for viruses with only a few immune-evasive substitutions (e.g., Beta, Gamma, and Delta), leading to relatively large antigenic distances. On the other hand, low titers may underestimate the antigenic distance for highly evasive viruses due to reaching the assay’s lower limit of detection. In agreement, the distance of the main cluster to Omicron BA.1 was larger in our map compared with the map by Wilks and colleagues. Nevertheless, these data indicate that human and hamster serum responses to SARS-CoV-2 infection are similar as they lead to topologically similar maps. However, the specific set of viruses and sera used to construct an antigenic map may influence antigenic distances, e.g., due to omission of sera against Omicron. As human sera post-primary infection are increasingly difficult to obtain, our data suggest that antigenic cartography using hamster antisera is a useful surrogate to assess antigenic relationships between SARS-CoV-2 variants.

The Omicron variant position in the map and limited cross-neutralization to the original cluster were corroborated by human post-vaccination antibody responses (Fig. 1). Omicron BA.1 and BA.2 were both poorly neutralized by human post-vaccination sera, which is in line with previous studies (*23*–*25*). The ameliorating effect of the booster vaccine on Omicron BA.2 responses suggests that population immunity against BA.2 may be sufficient to prevent flooding of healthcare systems and high levels of mortality, seen before for previous variants (*36*–*38*). In addition, the widespread circulation of BA.1 may lead to the broadening of the antibody response in previously infected or vaccinated individuals and subsequent dampening of the intensity of spread. However, in regions with low access to vaccines, a wave of primary infections with BA.1 would potentially lead to low level cross-protection and continued opportunity for further widespread circulation of BA.2 or other variants.

The limitations of this study include the lack of sufficient samples of human sera to validate the antigenic maps generated, however they closely resembled the map generated by Wilks and colleagues (*35*). It is also unclear whether the susceptibility of hamsters to newly emerging variants may vary and the generation of new antisera may not be possible. In addition, at the time of this study, hamster antisera generated against Omicron BA.2 were not available.

The antigenic cartography of SARS-CoV-2 visualizes clearly how BA.1 and BA.2 can both escape antibody responses without being antigenically similar. The emergence of both Omicron variants indicates that population immunity is selecting for SARS-CoV-2 variants that efficiently escape from neutralizing antibody responses, leading to the first signs of antigenic drift. SARS-CoV-2 will likely reach endemicity and cause annual or semiannual infection waves as seen for influenza and seasonal coronaviruses. Our study provides methods for the continuous monitoring of the antigenic evolution of SARS-CoV-2, which may inform the selection of vaccine strains for future use.

## MATERIALS AND METHODS

### Viruses and cell lines

HEK-293T cells were cultured in Dulbecco’s modified Eagle’s medium (DMEM) supplemented with 10% FCS, sodium pyruvate (1 mM, Gibco), non-essential amino acids (1x, Lonza), penicillin (100 IU/ mL), and streptomycin (100 IU/mL). VeroE6 cells were maintained in DMEM supplemented with 10% FCS, HEPES (20 mM, Lonza) and sodium pyruvate (1 mM), penicillin (100 IU/mL), and streptomycin (100 IU/mL). Calu-3 cells were maintained in Opti-MEM I (1×) + GlutaMAX (Gibco) supplemented with 10% FCS, penicillin (100 IU/mL), and streptomycin (100 IU/mL). All cell lines were kept at 37°C in a humidified CO_2_ incubator. Viruses propagated for infection in hamsters and neutralization assays were grown to passage 3 on Calu-3 cells (aside from 614G used in hamster inoculations, grown to passage 3 on Vero cells), harvested 48-72 hours post-infection, cleared for 5 min at 1000 × g, aliquoted and stored at -80°C until use. All work with infectious SARS-CoV-2 was performed in a Class II Biosafety Cabinet under BSL-3 conditions at Erasmus Medical Center.

### Pseudovirus production

Pseudoviruses were produced as described previously (*32*). Briefly, HEK-293T cells were transfected with pseudotyping vectors from InvivoGen (Original D614, D614G, Alpha, Beta, Kappa and Delta spike) or kindly provided by Dr. Berend Jan Bosch (Omicron spike). All spike expressing plasmids contained a deletion of the last 19 amino acids containing the Golgi retention signal of the SARS-CoV-2 spike protein. Twenty four hours post-infection cells were infected with pseudoviruses expressing VSV-G. Two hours post-infection, cells were washed three times with Opti-MEM I (1×) + GlutaMAX and replaced with medium containing anti-VSV-G neutralizing antibody (Absolute Antibody). The supernatant was collected after 24 and 48 hours, cleared by centrifugation at 2000 × g for 5 min, and stored at 4°C.

### Virus titrations

Stock titers were determined by preparing 10-fold serial dilutions in Opti-MEM I (1X) + GlutaMAX. One hundred μl of each dilution was added to monolayers of Calu-3 (or VeroE6 cells for 614G) cells in the same medium in a 12 well plate. After 4 hours at 37°C, cells were overlaid with 1.2% Avicel (FMC BioPolymer) in Opti-MEM I (1X) + GlutaMAX (Gibco) for 72 hours. Avicel was then removed and plates were fixed in formalin, permeabilized in 70% ethanol and washed in PBS. Cells were blocked in 3% BSA (bovine serum albumin; Sigma) in PBS, followed by rabbit anti-nucleocapsid (Sino biological; 1:2000) in PBS containing 0.1% BSA. Plates were washed in PBS then stained with goat anti-rabbit Alexa Fluor 488 (Invitrogen; 1:4000) in PBS containing 0.1% BSA. Plates were then washed again in PBS and scanned on the Amersham Typhoon Biomolecular Imager (channel Cy2; resolution 10 mm; GE Healthcare). Eight hour titers of SARS-CoV-2 and pseudoviruses were determined by preparing 10-fold serial dilutions in Opti-MEM I (1X) + GlutaMAX. Thirty μl of each dilution was added to monolayers of Calu-3 or VeroE6 cells in the same medium in a 96 well plate. After 16 hours at 37°C, Pseudovirus infected plates were fixed in paraformaldehyde and washed in PBS. After 8 hours at 37°C SARS-CoV-2 infected cells were fixed in formalin, permeabilized in 70% ethanol and washed in PBS. Cells were blocked in 3% BSA (bovine serum albumin; Sigma) in PBS, followed by rabbit anti-nucleocapsid (Sino biological; 1:2000) in PBS containing 0.1% BSA. Plates were washed in PBS then stained with goat anti-rabbit Alexa Fluor 488 (Invitrogen; 1:4000) in PBS containing 0.1% BSA. SARS-CoV-2 and Pseudovirus infected cells were next stained with Hoechst (ThermoFisher) and washed with PBS. Cells were imaged using the Opera Phenix spinning disk confocal HCS system (Perkin Elmer) equipped with a 10x air objective (NA 0.3) and 405 nm and 488 nm solid state lasers. Hoechst and GFP were detected using 435-480 nm and 500-550 nm emission filters, respectively. Nine fields per well were imaged covering approximately 50% of the individual wells. The number of green fluorescent protein (GFP) positive/ Alexa Fluor 488 positive infected cells were quantified using the Harmony software (version 4.9, Perkin Elmer).

### Hamster infections

Female Syrian golden hamsters (*Mesocricetus auratus*; 6 weeks old; Janvier, France) were handled in an ABSL-3 biocontainment laboratory. Groups of animals (n=4) were inoculated intranasally with 1.0x10^5 PFU (614G, Alpha, Beta, Gamma, Zeta, Mu) or 5.0x10^4 PFU (Delta, Omicron) in a total volume of 100μl per animal. Nasal washes (250μL) were taken at 7 dpi. All animals were sacrificed 26 dpi. Research involving animals was conducted in compliance with the Dutch legislation for the protection of animals used for scientific purposes (2014, implementing EU Directive 2010/63) and other relevant regulations. The licensed establishment where this research was conducted (Erasmus MC) has an approved OLAW Assurance # A5051-01. Research was conducted under a project license from the Dutch competent authority and the study protocol (#17-4312) was approved by the institutional Animal Welfare Body. Animals were housed in groups of 2 animals in filter top cages (T3, Techniplast), in Class III isolators allowing social interactions, under controlled conditions of humidity, temperature and light (12-hour light/12-hour dark cycles). Food and water were available ad libitum. Animals were cared for and monitored (pre- and post-infection) daily by qualified personnel. All animals were allowed to acclimatize to husbandry for at least 7 days. For unbiased experiments, all animals were randomly assigned to experimental groups. The animals were anesthetized (3-5% isoflurane) for all invasive procedures. Hamsters were euthanized by cardiac puncture under isoflurane anesthesia and cervical dislocation.

### Viral RNA quantification using qRT-PCR

RNA extraction was performed as described previously (*39*). Briefly, 60 μL of sample was lysed in 90 μL of MagNA Pure LC Lysis buffer (Roche) followed by a 15 min incubation with 50 μL Agencourt AMPure XP beads (Beckman Coulter). Beads were washed twice with 70% ethanol on a DynaMag-96 magnet (Invitrogen) and eluted in 50 μL diethylpyrocarbonate treated water. qRT-PCR was performed using primers targeting the E gene (*40*) and comparing the Ct values to a standard curve derived from a virus stock titrated on Calu-3 cells.

### Plaque reduction neutralization assay

Post-vaccination and post-Omicron BA.1 infection sera were obtained in the scope of the healthcare worker study performed at the Erasmus MC (*14*). This study was approved by the institutional review board of the Erasmus MC (medical ethical committee, MEC-2020-0264). All BNT162b2-vaccinated individuals had no prior infection with SARS-CoV-2. Sera from individuals infected with Omicron BA.1 was collected on average 25 days post symptom onset. PRNT50 assays were performed as described previously. Briefly, sera were heat inactivated for 30 min at 56°C. Sera were 3-fold serially diluted in 60 μL Opti-MEM I (1X) + GlutaMAX (Gibco). Four hundred PFU (based on 8 hours titrations) in 60 μL were added per well to a final volume of 120 μL and a serum dilution of 1:20 in the first well. Plates were incubated for 1 hour at 37°C. Next 100 μL of virus and serum mix was added to confluent monolayers of Calu-3 or VeroE6 cells. SARS-CoV-2 infected plates were incubated for 8 hours at 37°C before fixing in formalin and permeabilizing in ethanol. Plates were then washed in PBS and stained as described for virus titrations. Pseudovirus infected plates were incubated for 16 hours at 37°C before fixing in paraformaldehyde and washing in PBS. Nuclei were stained with Hoechst for 30 min. Cells were imaged using the Opera Phenix spinning disk confocal HCS system and the number of GFP-positive/Alexa Fluor 488 positive infected cells were quantified using the Harmony software as described above. The PRNT50 was calculated based on non-linear regression, followed by a Pearson correlation and linear regression analysis. All analyses were performed using GraphPad Prism 9 software.

### Illumina sequencing

Deep sequencing was performed as described previously (*14*). Briefly, RNA was extracted as described above followed by cDNA synthesis and PCR using the QIAseq® SARS-CoV-2 Primer Panel kit (Qiagen) according to the manufacturer’s protocol. Omicron samples were amplified with an additional 11 primers (as described by ARTIC V4.1 primer set). Amplicons were purified using 0.8x AMPure XP beads followed by converting 100ng of DNA to paired-end Illumina sequencing libraries using the KAPA HyperPlus library preparation kit (Roche). Samples were pooled and analyzed on an Illumina sequencer V3 MiSeq flow cell (2x300 cycles). The 614G virus used for hamster infections was cultured to passage 3 on Vero cells and its S protein did not contain additional mutations. All other variant were propagated to passage 3 on Calu-3 cells. For neutralization assays another passage 3 614G isolate (Bavpat-1) was used with an identical S amino acid sequence (European Virus Archive Global #026 V-03883) and another passage 3 Beta isolate with an identical S amino acid sequence. The 614G Bavpat-1 passage 3 sequence was identical to the passage 1 (kindly provided by Dr. Christian Drosten). The Alpha (B.1.1.7), Gamma (P.1), Delta (B.1.617.2), Delta AY.4.2 (B.1.617.2 AY.4.2), Lambda (C.37), Mu (B.1.621), Omicron BA.1 (B.1.1.529 BA.1) and Omicron BA.2 (B.1.1.529 BA.2) variant passage 3 sequences were identical to the original respiratory specimens. Low coverage regions in spike were confirmed by Sanger sequencing. The Beta variant (B.1.351, used for hamster inoculations) passage 3 sequence contained one synonymous mutation compared to the original specimen: A26449C (Wuhan-Hu-1 position) in E. The Beta variant (B.1.351, used in neutralization assays) passage 3 sequence contained two mutations compared the original respiratory specimen: one synonymous mutation C13860T (Wuhan-Hu-1 position) in ORF1ab and a L71P change in the E gene (T26456C, Wuhan-Hu-1 position). The spike changes of all variants compared to Wuhan-Hu-1 are indicated in Fig. S2. All isolate sequences were submitted to GenBank. Hamster nasal wash sample sequences were identical to the input viruses.

### Antigenic cartography

Antigenic map construction was performed as described previously (*31*). Briefly, antigenic cartography is a method to quantify and visualize neutralization data. In an antigenic map, the distance between antiserum point S and antigen point A corresponds to the difference between the log2 of the maximum titer observed for antiserum S against any antigen and the log2 of the titer for antiserum S against antigen A. Thus, each titer in a cross-titration can be thought of as specifying a target distance for the points in an antigenic map. Modified multidimensional scaling methods are then used to arrange the antigen and antiserum points in an antigenic map to best satisfy the target distances specified by the neutralization data. The result is a map in which the distance between the points represents antigenic distance as measured by the neutralization assay in which the distances between antigens and antisera are inversely related to the log2 titer. Because antisera are tested against multiple antigens, and antigens tested against multiple antisera, many measurements can be used to determine the position of the antigen and antiserum in an antigenic map, thus improving the resolution of the data. The antigenic maps were computed with the Racmacs package (https://acorg.github.io/Racmacs/, version 1.1.18.) in R. A web-based version of the software is available from https://www.antigenic-cartography.org/ . The maps were constructed using 1000 optimizations, with the minimum column basis parameter set to “none”.

### Statistical analysis

Statistical analysis was performed with the GraphPad Prism 9 software using a one-way ANOVA followed by a Bonferroni multiple-comparison test.

## Supplementary Materials

### Other Supplementary Material for this manuscript includes the following:

MDAR Reproducibility Checklist
