# Supplementary material for: Antigenic cartography of SARS-CoV-2 reveals that Omicron BA.1 and BA.2 are antigenically distinct
Source: Sci Immunol. 2022 Jun 23:eabq4450. doi: 10.1126/sciimmunol.abq4450 (PMC9273038; doi:10.1126/sciimmunol.abq4450)
Supplement: Supplementary file 1 — Figs. S1 to S10 Tables S1 and S2 [file sciimmunol.abq4450_sm.pdf]

Science Immunology
AAAS

Supplementary Materials for

Antigenic cartography of SARS-CoV-2 reveals that Omicron BA.1 and BA.2 are antigenically distinct

Anna Z. Mykytyn *et al.*

Corresponding author: Bart L. Haagmans, b.haagmans@erasmusmc.nl

DOI: 10.1126/sciimmunol.abq4450

The PDF file includes:

Figs. S1 to S10
Tables S1 and S2

Other Supplementary Material for this manuscript includes the following:

MDAR Reproducibility Checklist
Data file S1

Antigenic cartography of SARS-CoV-2 reveals that Omicron BA.1 and BA.2 are antigenically distinct

Authors: Anna Z. Mykytyn[1], Melanie Rissmann[1]†, Adinda Kok[1]†, Miruna E. Rosu[1]†, Debby Schipper[1], Tim I. Breugem[1], Petra B. van den Doel[1], Felicity Chandler[1], Theo Bestebroer[1], Maurice de Wit[2], Martin E. van Royen[3], Richard Molenkamp[1], Bas B. Oude Munnink[1], Rory D. de Vries[1], Corine GeurtsvanKessel[1], Derek J. Smith[4], Marion P. G. Koopmans[1], Barry Rockx[1], Mart M. Lamers[1], Ron Fouchier[1], Bart L. Haagmans[1]*

Supplementary Materials:

Figures S1-S10

Tables S1-S2

Data file S1 (raw data file; Excel spreadsheet)

MDAR Reproducibility Checklist

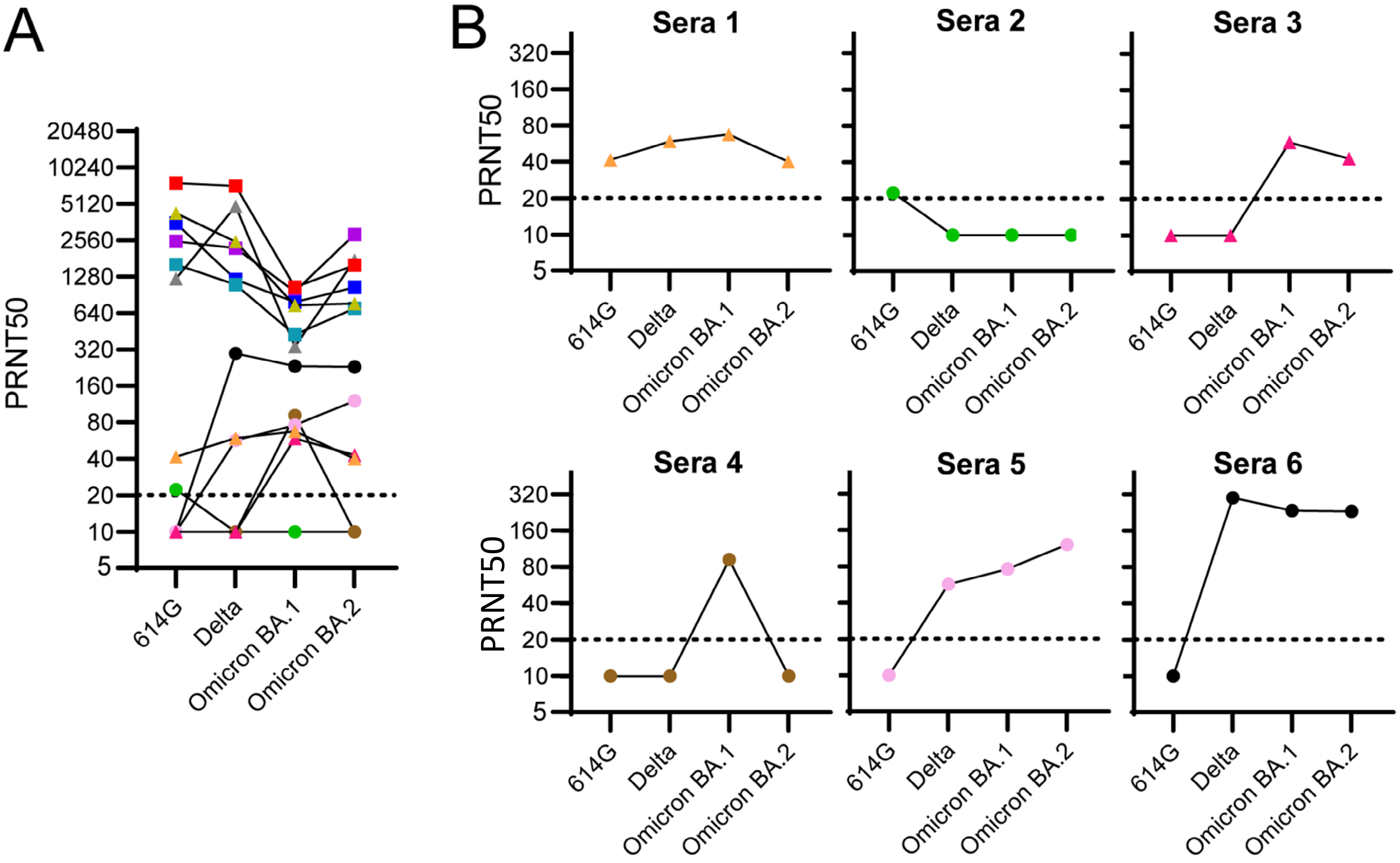

Fig. S1. Neutralizing activity of human sera post-Omicron BA.1 infection. (**A**) Neutralization titers against 614G, Delta, Omicron BA.1 and Omicron BA.2 after primary Omicron BA.1 infection.(**B**) Individual sera neutralization titers against 614G, Delta, Omicron BA.1 and Omicron BA.2 from 6 lowest responders in **A.** PRNT50: plaque reduction neutralization titers resulting in 50% plaque reduction. Dotted lines indicate limits of detection. Circles denote individuals without prior infection, squares denote individuals with a prior infection and triangles denote individuals sampled twice (the first sample being the yellow and orange triangles and the second sample being the grey and fuchsia triangles, respectively).

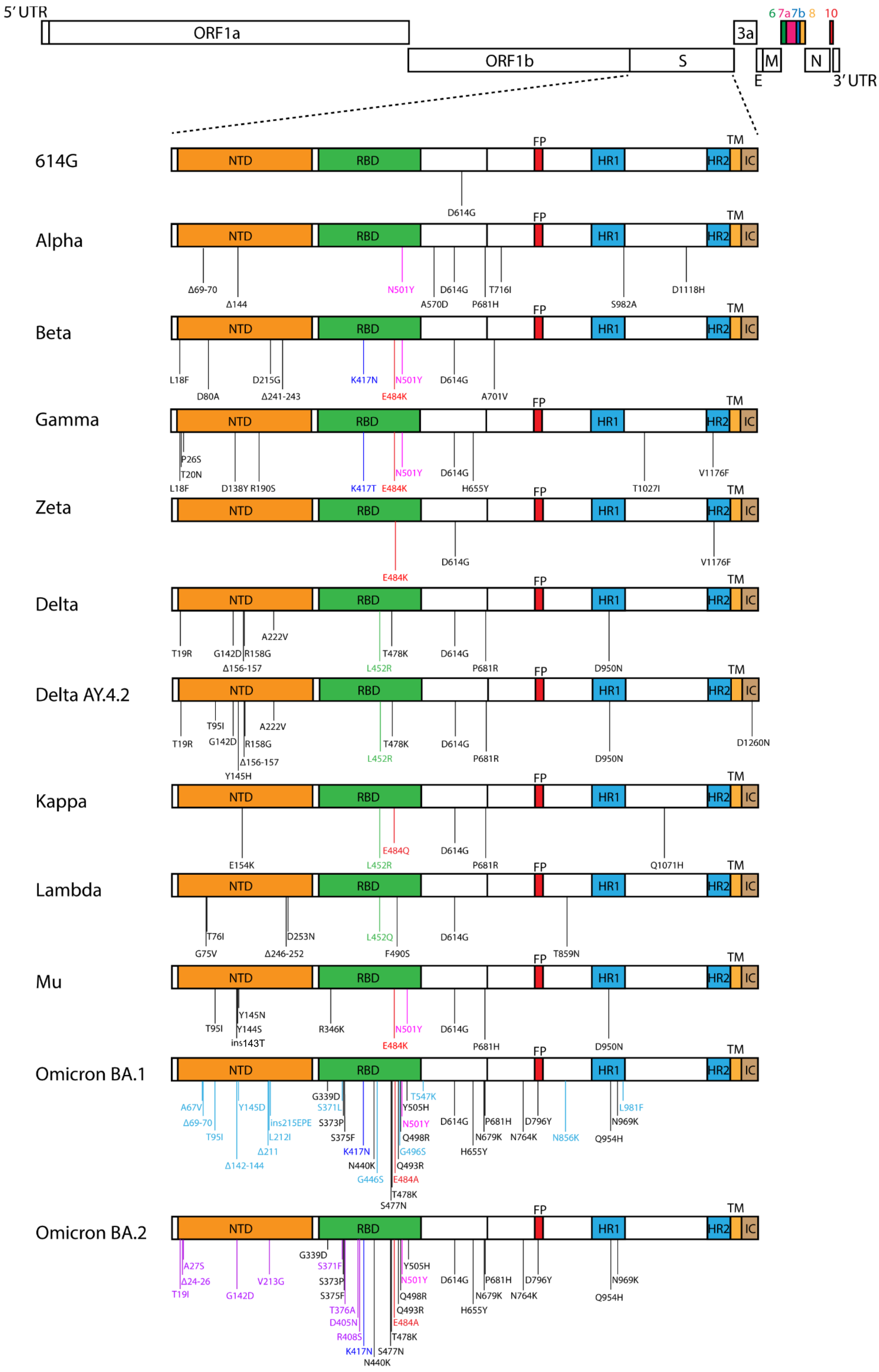
5′ UTR
ORF1a
ORF1b
S
3a
6 7a 7b 8
10
E
M
N
3′ UTR
614G
NTD
RBD
FP
HR1
TM
HR2
IC
D614G
Alpha
Δ69-70
Δ144
N501Y
A570D
D614G
P681H
T716I
S982A
D1118H
Beta
L18F
D80A
D215G
Δ241-243
K417N
E484K
N501Y
D614G
A701V
Gamma
L18F
T20N
P26S
D138Y
R190S
K417T
E484K
N501Y
D614G
H655Y
T1027I
V1176F
Zeta
E484K
D614G
V1176F
Delta
T19R
G142D
Δ156-157
R158G
A222V
L452R
T478K
D614G
P681R
D950N
Delta AY.4.2
T19R
T95I
G142D
Y145H
Δ156-157
R158G
A222V
L452R
T478K
D614G
P681R
D950N
D1260N
Kappa
E154K
L452R
E484Q
D614G
P681R
Q1071H
Lambda
G75V
T76I
Δ246-252
D253N
L452Q
F490S
D614G
T859N
Mu
T95I
Y144S
Y145N
ins143T
R346K
E484K
N501Y
D614G
P681H
D950N
Omicron BA.1
A67V
Δ69-70
T95I
Δ142-144
Y145D
Δ211
L212I
ins215EPE
G339D
S371L
S373P
S375F
K417N
N440K
G446S
S477N
T478K
E484A
Q493R
G496S
Q498R
N501Y
Y505H
T547K
D614G
H655Y
N679K
P681H
N764K
D796Y
N856K
Q954H
N969K
L981F
Omicron BA.2
T19I
Δ24-26
A27S
G142D
V213G
G339D
S371F
S373P
S375F
T376A
D405N
R408S
K417N
N440K
S477N
T478K
E484A
Q493R
Q498R
N501Y
Y505H
D614G
H655Y
N679K
P681H
N764K
D796Y
Q954H
N969K

Fig. S2. Spike substitutions in SARS-CoV-2 variants. Indicated are Spike substitutions relative to Wuhan-Hu-1 present in all variants assessed. Indicated in red are substitutions at the 484 position, in pink substitutions at the 501 position and in dark blue substitutions at the 417 position. Differential substitutions between Omicron BA.1 and Omicron BA.2 are indicated in light blue and purple respectively.

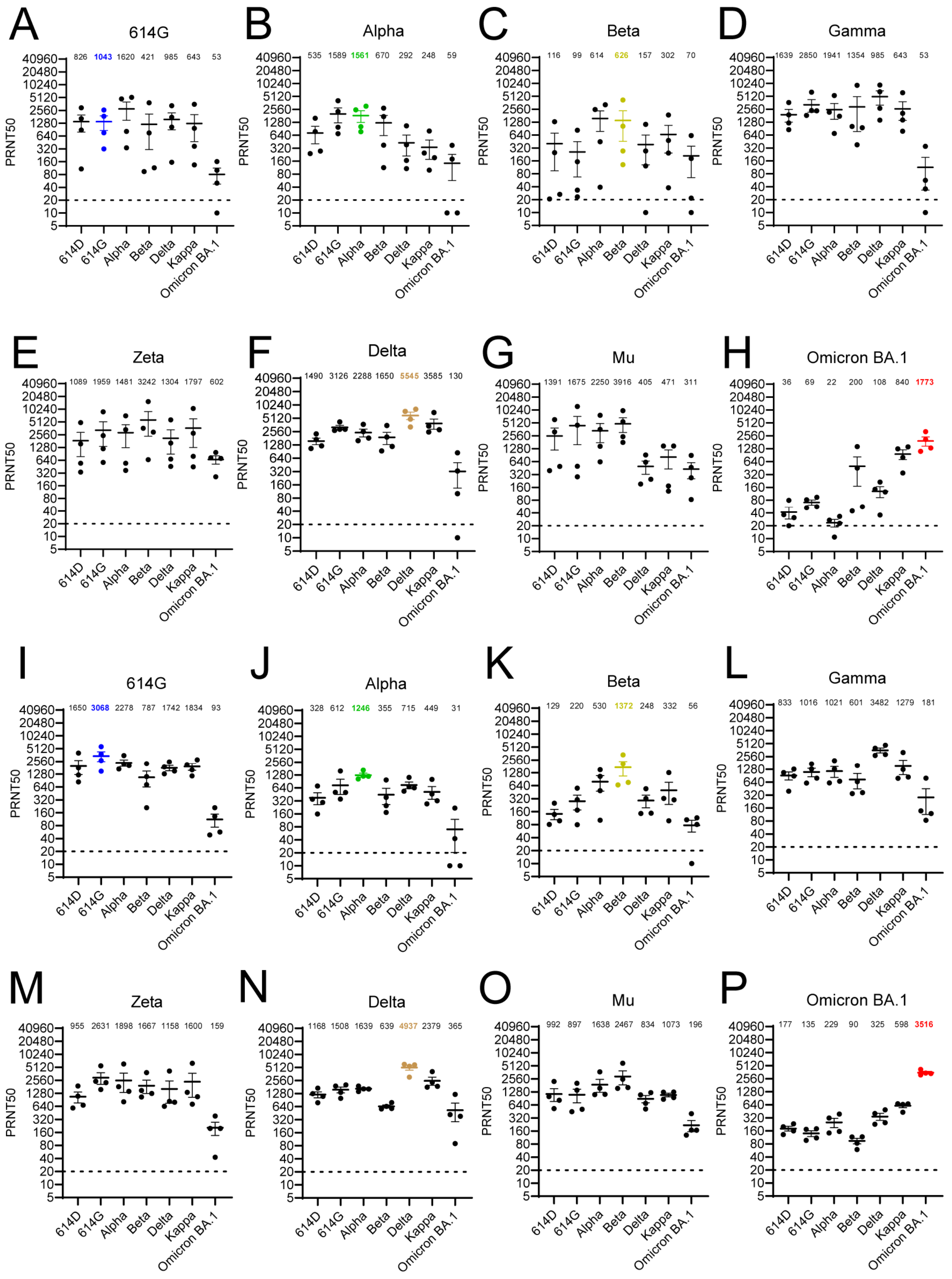

Fig. S3. Pseudotyped SARS-CoV-2 neutralization titers. (**A-H**) Neutralizing titers against pseudotyped SARS-CoV-2 on VeroE6 cells of hamsters infected with either (**A**) 614G, (**B**) Alpha, (**C**) Beta, (**D**) Gamma, (**E)** Zeta, (**F**) Delta, (**G**) Mu or (**H**) Omicron BA.1. (**I-P**) Neutralizing titers against pseudotyped SARS-CoV-2 on Calu-3 cells of hamsters infected with either (**I**) 614G, (**J**) Alpha, (**K**) Beta, (**L**) Gamma, (**M)** Zeta, (**N**) Delta, (**O**) Mu or (**P**) Omicron BA.1. Geometric mean is displayed above each graph. PRNT50: plaque reduction neutralization titers resulting in 50% plaque reduction. Dotted lines indicate limits of detection. Error bars indicate SEM.

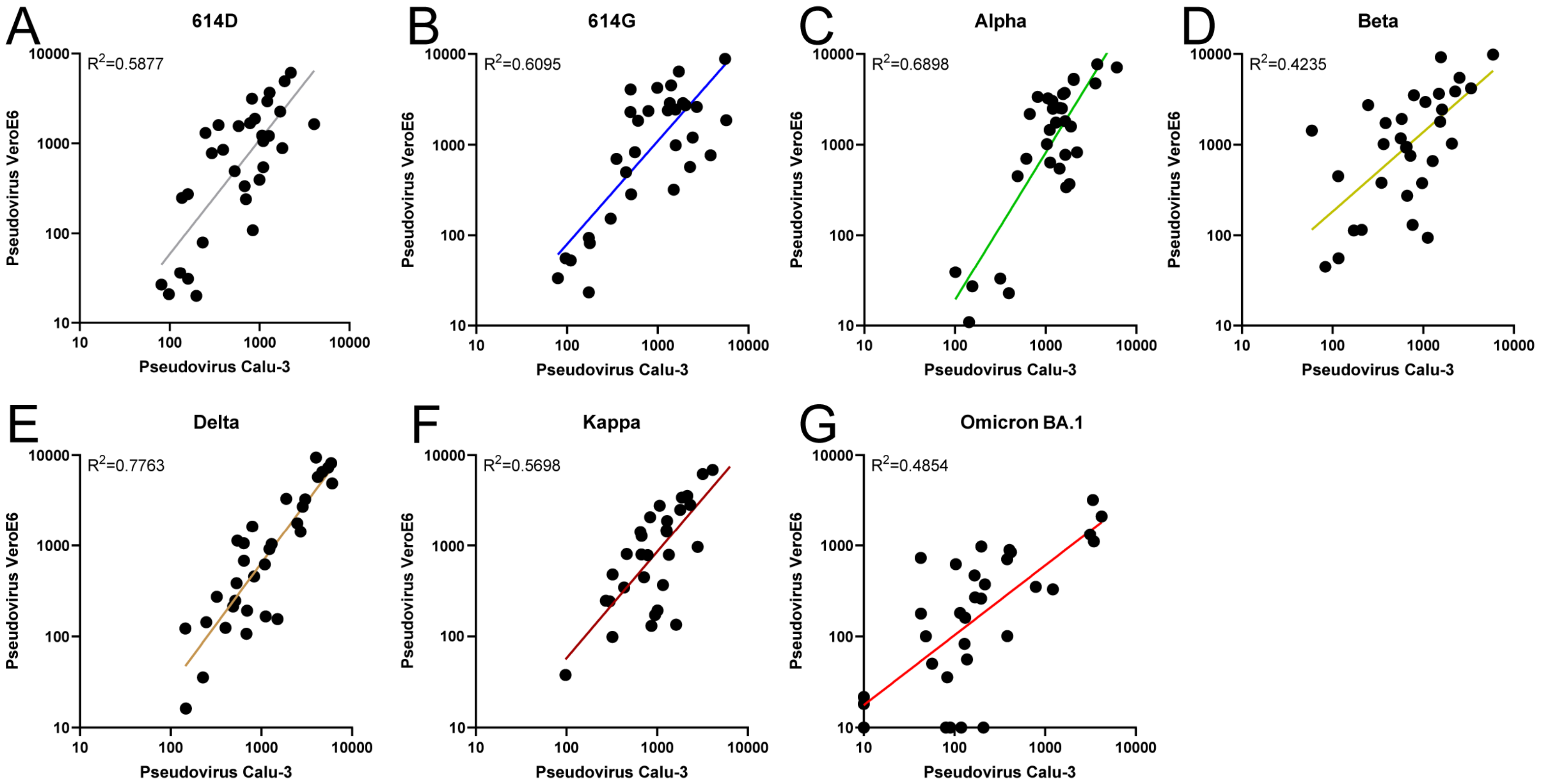

Fig. S4. SARS-CoV-2 pseudovirus neutralization titers generated on VeroE6 cells correlate with titers generated on Calu-3 cells. (**A-G**) Correlation of PRNT50 titers obtained with pseudotyped (**A**) 614D, (**B**) 614G, (**C**) Alpha, (**D**) Beta, (**E)** Delta, (**F**) Kappa and (**G**) Omicron BA.1 variants on VeroE6 cells compared to Calu-3 cells. PRNT50: plaque reduction neutralization titers resulting in 50% plaque reduction. Linear regression line is depicted in each panel.

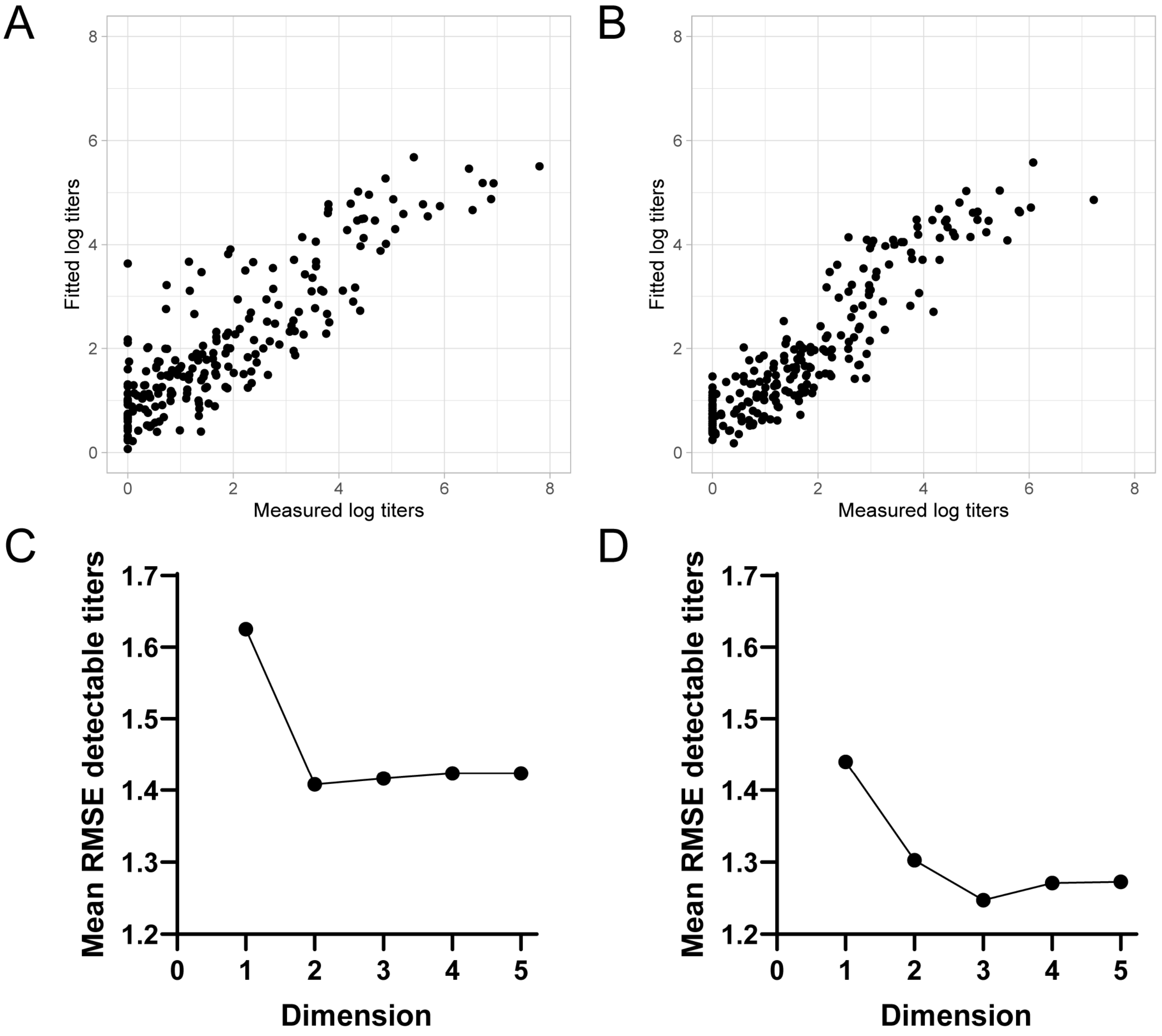

Fig. S5. Validation of representing neutralization data in two dimensional antigenic maps. (**A-B**) Scatter plot of detectable neutralizing titers on the x-axis and fitted titers from the antigenic maps on the y-axis of maps generated in Fig. 3 and 4. Scatter plots generated on data obtained with pseudovirus neutralizations on (**A**) VeroE6 cells (Fig. 3A) and (**B**) Calu-3 cells. (**C-D**) Dimensionality tests indicating the mean RMSE of detectable neutralization titers in 1 to 5 dimensions for maps generated with data from neutralization on (**C**) VeroE6 cells or (**D**) Calu-3 cells. For each dimension 100 antigenic maps with 1000 optimizations each are generated while randomly excluding 10% of the titers. The mean RMSE is calculated by comparing the predicted titers in each run to the actual measured titers on a log2 scale. RMSE: root mean square prediction error.

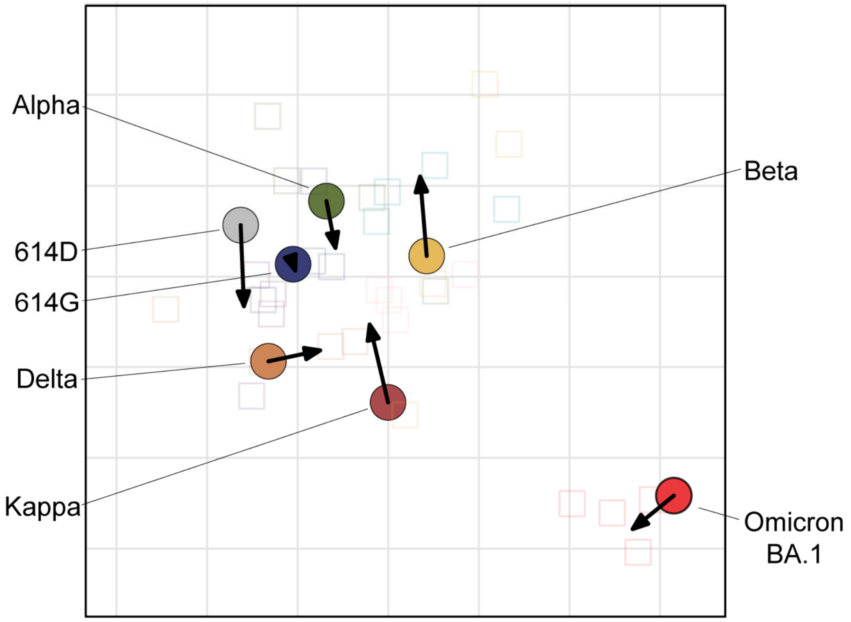

Fig. S6. Comparison of the antigenic map generated with pseudovirus on VeroE6 cells to Calu-3 cells. Antigenic map from Fig. 3A with arrows indicating position of antigens in antigenic map in Fig. 3B. See legend to Fig. 3 for details.

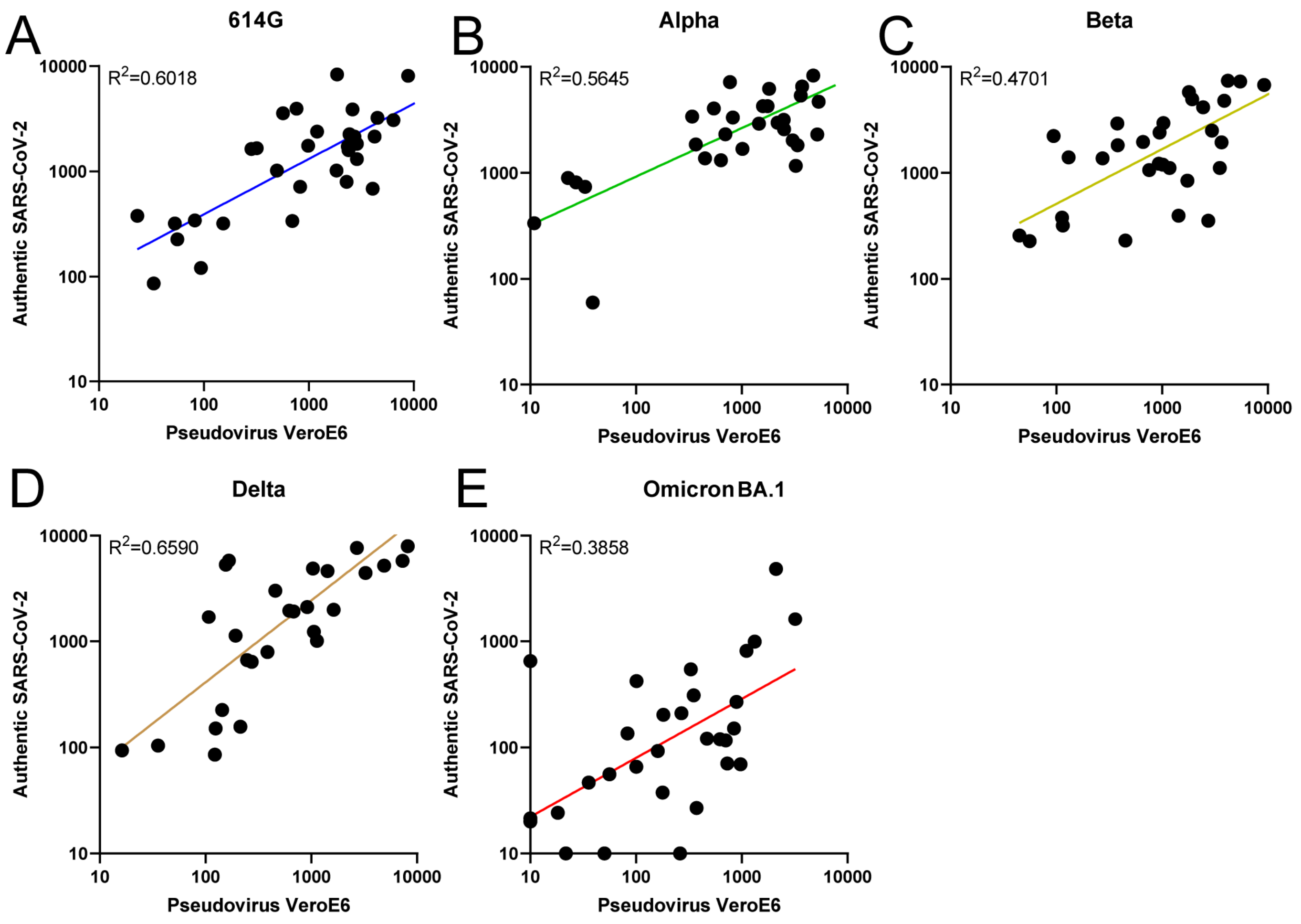

Fig. S7. SARS-CoV-2 pseudovirus neutralization titers on VeroE6 cells correlate with authentic SARS-CoV-2 neutralization titers. (**A-E**) Correlation of PRNT50 titers obtained with pseudotyped and authentic SARS-CoV-2 (**A**) 614G, (**B**) Alpha, (**C**) Beta, (**D**) Delta and (**E**) Omicron variants on VeroE6 cells. PRNT50: plaque reduction neutralization titers resulting in 50% plaque reduction. Linear regression line is depicted in each panel.

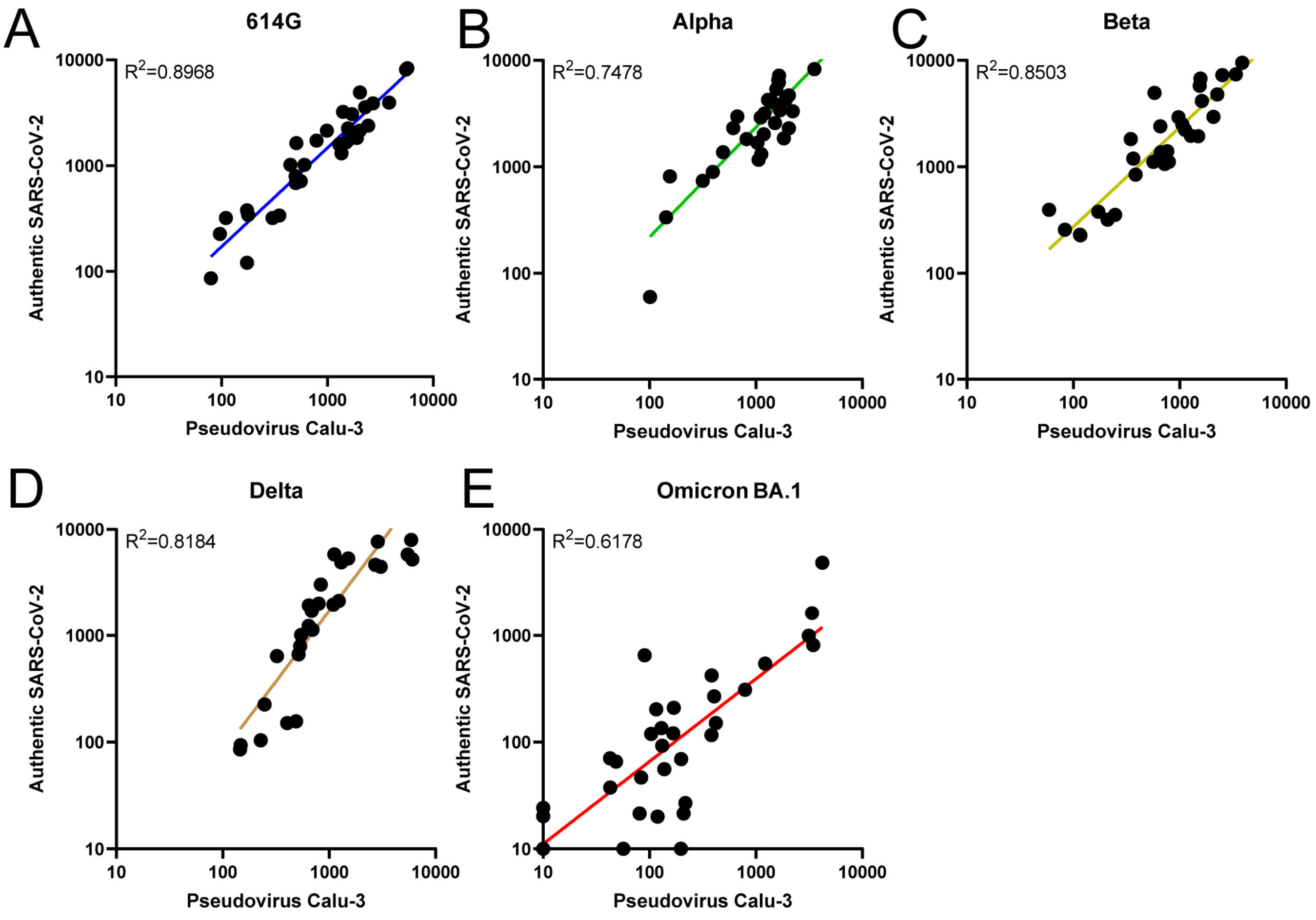

Fig. S8. SARS-CoV-2 pseudovirus neutralization titers on Calu-3 cells correlate with authentic SARS-CoV-2 neutralization titers. (**A-E**) Correlation of PRNT50 titers obtained with pseudotyped and authentic SARS-CoV-2 (**A**) 614G, (**B**) Alpha, (**C**) Beta, (**D**) Delta and (**E**) Omicron variants on Calu-3 cells. PRNT50: plaque reduction neutralization titers resulting in 50% plaque reduction. Linear regression line is depicted in each panel.

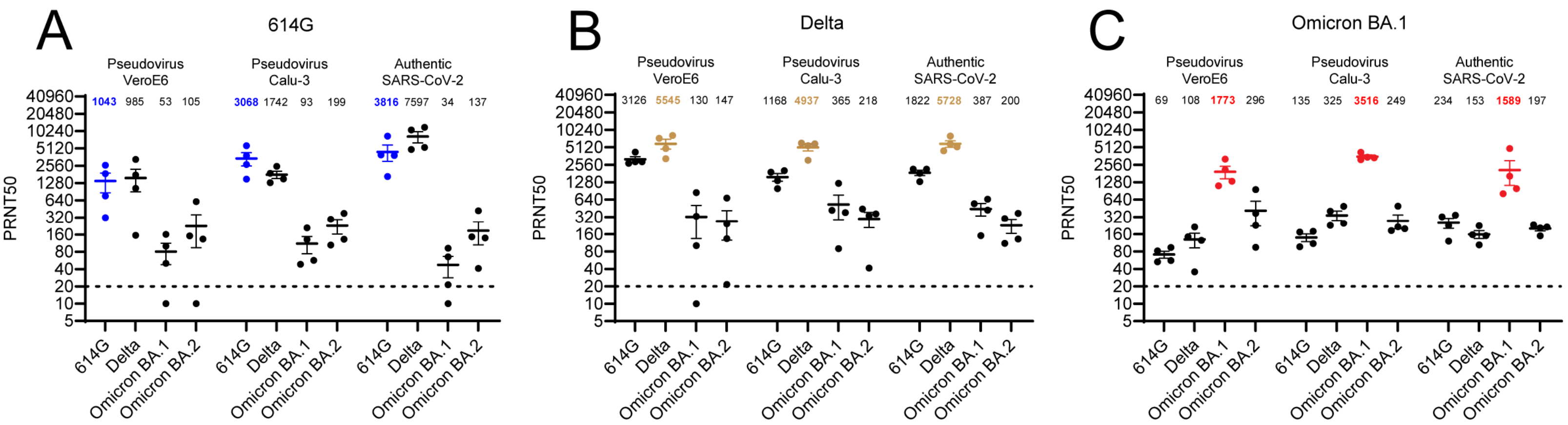

Fig. S9. Authentic and pseudotyped SARS-CoV-2 neutralization titers of Omicron BA.1 and BA.2. (A-C) Neutralizing titers of hamsters infected with (A) 614G, (B) Delta and (C) Omicron BA.1 against 614G, Delta, Omicron BA.1 and BA.2 authentic and pseudotyped viruses. Neutralizing titers generated using authentic SARS-Cov-2 are a re-display of data in Fig. 4. Neutralizing titers generated against pseudotyped 614G, Delta and Omicron BA.1 viruses are a re-display of data in Fig. S3. Geometric mean is displayed above each graph. PRNT50: plaque reduction neutralization titers resulting in 50% plaque reduction. Dotted lines indicate limits of detection. Error bars indicate SEM.

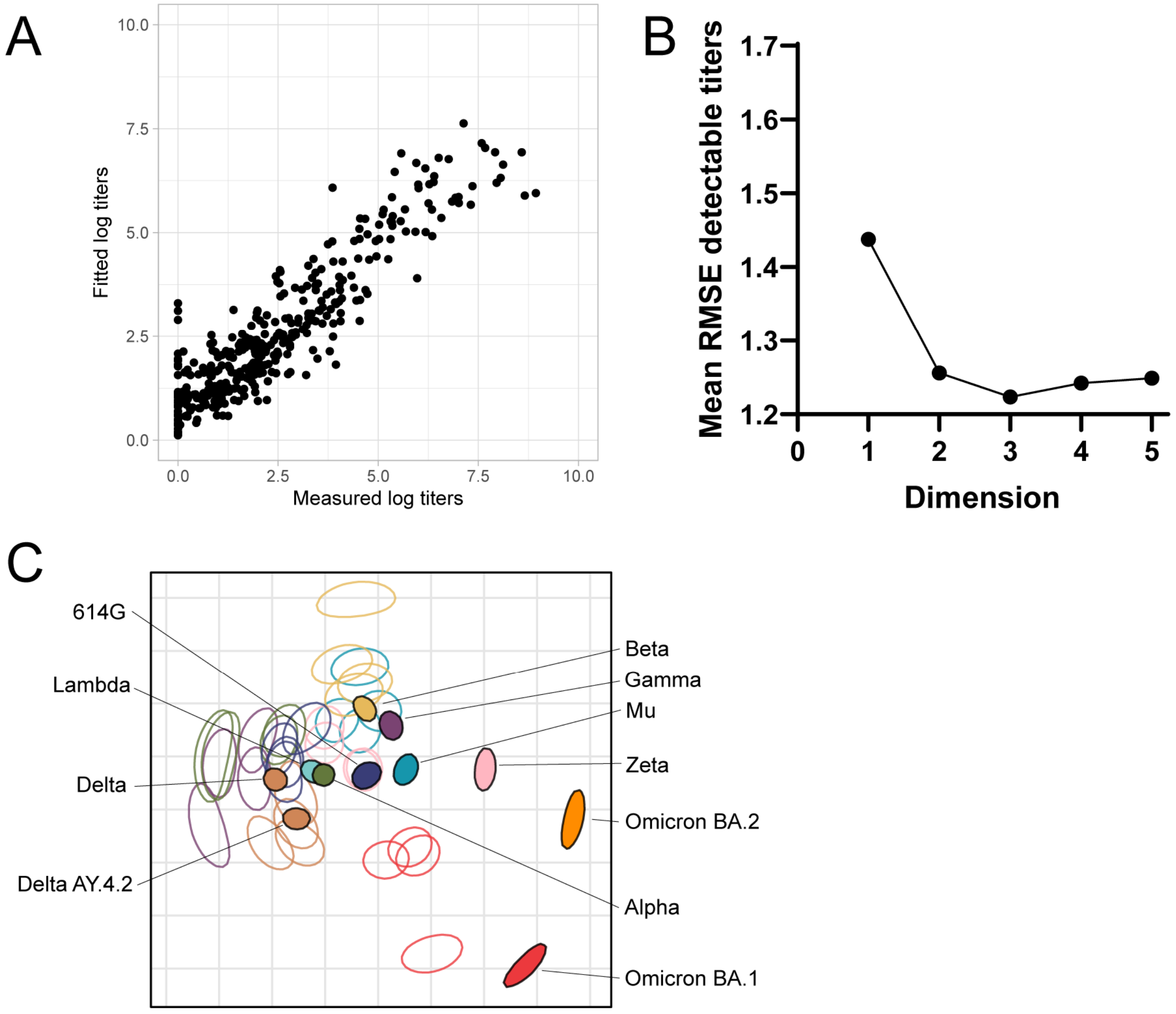

Fig. S10. Uncertainty in antigen and antisera positions of antigenic map generated with authentic SARS-CoV-2. (**A**) Scatter plot of detectable neutralizing titers on the x-axis and fitted titers from the antigenic map on the y-axis of map generated in Fig. 4I. (**B**) Dimensionality test indicating the mean RMSE of detectable neutralization titers in 1 to 5 dimensions for antigenic map generated in Fig. 4I. See Fig. S4 for details. (**C**) Antigenic map from Fig. 4I with depicted regions (triangulation blobs) in which a particular antigen or antisera can be positioned without increasing the total stress of the map above one unit. See Fig. 3 legend for details.

Table S1. Demographic data of BNT162b2-vaccinated individuals

**Male**	4/10 (40%)
**Female**	6/10 (60%)
**Age**<br>**(mean years + SD)**	42.7 ± 14.5
**Prior infection status**	0/10 (0%)
**Days post 1st vaccination and serum collection**<br>**(mean + SD)**	20 ± 1.6
**Days post 2nd vaccination and serum collection**<br>**(mean + SD)**	26 ± 3.0
**Days post 3rd vaccination and serum collection**<br>**(mean + SD)**	38 ± 2.3

Table S2. Demographic data of primary Omicron BA.1-infected individuals

**Male**	2/10 (20%)
**Female**	8/10 (80%)
**Age**<br>**(mean years + SD)**	36 ± 11.2
**Prior infection status**	4/10 (40%)
**Days post positive PCR**<br>**(mean + SD)**	25 ± 8.1
**Days post symptom onset**	23 ± 1.7 (7/10)<br>(3/10 unknown)
